# Comorbidity Classification from Clinical Free-Text using Large Language Models: Application to Sleep Disorder Patients

**DOI:** 10.1007/s10916-026-02343-y

**Published:** 2026-02-19

**Authors:** Yihan Deng, Fabio Dennstädt, Irina Filchenko, Julia van der Meer, Xiaoli Yang, Markus H. Schmidt, Claudio L. A. Bassetti, Athina Tzovara, Kerstin Denecke

**Affiliations:** 1 Department Engineering and Computer Science, Institute Patient-centered Digital Health, Bern University of Applied Sciences, Quellgasse 21, Biel, 2502 Switzerland; 2 Department of Radiation Oncology, University Hospital and University of Bern, Inselspital, Freiburgstrasse 18, Bern, 3010 Bern Switzerland; 3 Department of Neurology, Inselspital, University Hospital and University of Bern, Freiburgstrasse 16, Bern, 3010 Bern Switzerland; 4 Department of Pulmonary Medicine, Allergology and Clinical Immunology, Inselspital Bern, Freiburgstrasse 20, Bern, 3010 Bern Switzerland; 5 Department of Informatics, University of Bern, Hochschulstrasse 6, Bern, 3010 Bern Switzerland

**Keywords:** Comorbidity extraction, Sleep diagnostics, Large language model, LLM, Prompt-based classification

## Abstract

Patients presenting to neurology clinics commonly have a complex history of comorbidities and partially documented health trajectories, making it essential to reliably extract comorbidity information from historical records. However, existing extraction methods, ranging from rule-based systems to classical machine learning (ML), often have limited accuracy, scalability, or adaptability across diverse documents. We present a large language model (LLM)–based framework for comorbidity extraction from diagnostic texts, capable of handling various prompt formats and textual sources such as patient history, comorbidities, and sleep assessments. The instruction fine-tuned Mistral-24B (Instruct-2501) model achieves 95% macro classification accuracy and 92% F1 score across six common classes of comorbidities, achieving strong performance that is competitive with metrics reported in prior clinical phenotyping and information extraction studies, while complementing recent transformer-based clinical NLP frameworks. The proposed method extracts comorbidities through a transparent hierarchical approach, thereby supporting clinical analysis and providing interpretable insights for disease modeling and personalized treatment planning in sleep medicine.

## Introduction

Information on comorbidities in patients with sleep–wake disorders is often incomplete and inconsistently documented. Such information is frequently confined to unstructured free-text sources, including patient histories, sleep reports, and diagnostic notes. Moreover, comparable comorbidities are described across multiple classification systems–such as the International Classification of Diseases (ICD) [1], the Diagnostic and Statistical Manual of Mental Disorders (DSM), and the American Academy of Sleep Medicine (AASM) International Classification of Sleep Disorders, Third Edition [2], leading to additional heterogeneity in documentation. To date, only limited solutions exist for the systematic coding of diseases within routine clinical workflows and their integration into electronic medical information systems (e.g., EPIC). However, a substantial amount of clinically valuable information remains in historical records, which are often only available as plain text. This unstructured nature complicates automated extraction. Traditional approaches, including rule-based systems and conventional machine learning (ML) methods [3–5] can identify explicit disease mentions in text but often struggle with linguistic variability and contextual nuances–such as distinguishing between current and past conditions or specifying subtypes of a disorder [6, 7]. Recent advances in large language models (LLMs) have shown promise in addressing these challenges [8]. Models such as BioBERT [9], ClinicalBERT [10], and PubMedBERT [11] were successfully applied to biomedical text mining and extraction of clinical information. More recent general-purpose LLMs, including Mistral-24B (Instruct-2501) [12], extend these capabilities by handling diverse document styles and supporting few-shot learning. In this study, we demonstrate the potential of a fine-tuned Mistral-24B (Instruct-2501) model to extract comorbidities from unstructured diagnostic reports in the context of sleep disorders.

By moving beyond surface mentions in text and capturing both broad categories and clinically relevant subtypes, the approach moves beyond surface-level mentions in text and demonstrates performance comparable to, or exceeding, metrics reported in prior clinical phenotyping studies, while enabling richer contextual interpretation.

### Advances in Language Models for Processing Data from Sleep Medicine

Over the past five years, advances in natural language processing (NLP) considerably improved the processing of clinical text, with emerging applications in sleep medicine. Early work in this domain predominantly relied on rule-based methods and classical ML to extract key sleep parameters (e.g., total sleep time, sleep efficiency, apnea–hypopnea index) from polysomnography (PSG) reports. For instance, Rahman et al. reported F1-scores exceeding 90% for extracting sleep metrics from PSG impressions [13]. These systems laid the groundwork for structuring traditional sleep study findings. More recently, transformer-based and LLM approaches have demonstrated improved capabilities in extracting nuanced clinical concepts. For example, Sivarajkumar et al. [14] compared rule-based methods, classical ML, and a fine-tuned LLaMA2 model for identifying sleep-related entities (e.g., snoring, insomnia) in clinical notes. The fine-tuned LLM achieved high precision comparable to rule-based systems, although its performance was sensitive to dataset size. These findings suggest that while rule-based methods remain effective for well-defined extraction tasks, transformer-based models offer greater flexibility for more complex clinical text understanding.

### LLMs and Integrative Approaches for Sleep Diagnostics and Comorbidity Extraction

LLMs such as GPT-4, along with domain-specialized transformer models like BioBERT, considerably advanced NLP applications in sleep medicine. Zhou et al. (2025) introduced a hybrid pipeline combining a Dynamic Seagull Search algorithm with an LLM classifier to detect sleep-disordered breathing from multimodal electronic health records’ inputs, achieving nearly 99% accuracy in identifying obstructive sleep apnoe (OSA) [15]. Similarly, Ahmed et al. [16] employed BioClinicalBERT and BlueBERT to “deep phenotype” OSA and its comorbidities from discharge summaries, leveraging contextual embeddings to expand disease lexicons and achieving weighted AUCs of 0.9 for comorbidity detection and 0.86 for mortality prediction. Accurate identification of comorbidities is critical, as patients with sleep disorders frequently present with overlapping cardiovascular and metabolic conditions. Traditional NLP pipelines often combine named entity recognition (NER) with relation extraction, and transformer-based models such as fine-tuned BERT have shown strong performance in detecting common comorbidities such as hypertension and diabetes [17]. Rule-based phenotyping methods, such as combining diagnosis codes with text mining, have further confirmed higher prevalence of hypertension, diabetes, dyslipidemia, and stroke among OSA patients [18]. More recently, generative LLMs demonstrated the ability to move beyond entity detection and interpret entire patient profiles. In a pilot study, Seifen et al. (2025) evaluated ChatGPT (GPT-4) on simulated sleep patient profiles including demographics, lab values, and diagnoses. The model achieved perfect agreement on smoking cessation recommendations, over 90% agreement on weight loss and endocrine evaluations, and reliably flagged comorbidities such as hypertension and metabolic risk, though it occasionally over-recommended ancillary testing [19]. These findings suggest that LLMs can support triage, risk stratification, and decision-making in sleep clinics when carefully directed. LLMs also offer practical advantages for multimorbidity research by extracting rich patient information from unstructured notes without extensive training data. Zhang et al. (2025) [20] demonstrated that a single LLM framework could accurately retrieve diagnoses, lifestyle details, symptom history, and medications with F1 scores of 95–99%, effectively acting as an automated chart reviewer. Such systems can be flexibly extended to include emerging comorbidities relevant to sleep medicine–for example, cognitive impairment or glaucoma–making them a versatile tool for both research and clinical workflows. To enable such applications, comorbidity extraction systems must ingest heterogeneous data sources. In the Bern Sleep–Wake Registry (BSWR), polysomnography (PSG), multiple sleep latency test (MSLT), and wrist actigraphy (AWT) reports all contain substantial narrative components alongside quantitative measures. While these modalities provide complementary clinical information–ranging from diagnostic impressions and result interpretations to structured sleep metrics–the degree of narrative content varies across report types and cannot be assumed to be lower in MSLT or actigraphy reports than in PSG reports. Annotated sleep stage sequences (hypnograms) are emerging as another textual data vector [14], though still exploratory. Free-text patient histories and clinical notes remain rich sources, as demonstrated by Sivarajkumar et al., who successfully extracted lifestyle factors and medical histories even when sleep-specific terms were sparse [14]. Integrative approaches that combine structured and unstructured data are becoming more common. Ahmed et al. [16] demonstrated that a hybrid pipeline–first assigning structured ICD codes and then augmenting them with LLM-driven lexicon expansion–enhanced comorbidity detection. Likewise, Zhou et al. [15] incorporated comorbidity features into their OSA classifier, illustrating the value of multi-modal integration in improving diagnostic accuracy.

### Contribution of this Work

In summary, the landscape of comorbidity extraction has evolved from rule-based phenotyping and basic machine learning toward sophisticated, multi-modal transformer-based systems [20, 21]. LLMs (e.g., GPT-4, BioClinicalBERT) are demonstrating strong performance in both entity recognition and comorbidity detection. Nonetheless, challenges remain in addressing multi-label classification and capturing the full spectrum of comorbidities in complex sleep patient profiles.

This work builds on this trajectory by introducing a fine-tuned Hugging Face language-model–based framework (such as Mistral-24B (Instruct-2501)) that performs robust binary classification across multiple comorbidities and integrates both structured and unstructured sleep diagnostic text. Our contributions are threefold:

1. We design and evaluate a hierarchical, multi-label classification scheme for sleep-related comorbidities that combines top-level and subtype decisions, and explicitly quantifies hierarchical consistency.
2. We instantiate this scheme in a LangGraph-based multi-agent architecture that yields modular, auditable decision paths aligned with clinically meaningful categories.
3. We demonstrate strong performance on a curated sleep-medicine dataset, with results that compare favorably to metrics reported in prior rule-based, classical machine learning, and transformer-based clinical NLP studies, and provide a preliminary cross-institution benchmark on MIMIC-IV discharge summaries to explore generalizability.

## Dataset

The dataset used in this study originates from a cohort of inpatients treated at the Sleep-Wake-Epilepsy Center of the Inselspital, University Hospital Bern, between 2000 and 2021. Secondary use of the Bern Sleep-Wake Registry was approved by the Cantonal Ethics Committee (KEK-Nr. 2022-00415: “Bern Sleep Registry: the sleep disorder patient cohort of the Inselspital, University Hospital Bern”).

As of 2024, the Bern Sleep–Wake Registry comprises 11,855 patients who met the general inclusion criteria. For the present comorbidity classification study, we further restricted the cohort to patients with available free-text diagnostic documentation in the clinical information system (IPDOS), resulting in a final study population of 3,690 patients.

In the Bern Sleep–Wake Registry, free-text descriptions of diagnoses are available from IPDOS. Each polysomnography (PSG) recording additionally provides free-text information on the patient’s clinical history.

The database contains de-identified clinical records of patients diagnosed with sleep disorders. Most historic records were retrospectively classified according to ICSD-3 (International Classification of Sleep Disorders, Third Edition) [22] criteria by clinical experts. For newer records, ICSD-3 annotations were either manually entered per patient and event in REDCap [23], or provided at the level of PSG tests based on test outcomes. In the latter case, annotations may not always capture the full range of markers and symptoms required for a comprehensive ICSD-3 classification. The Bern Sleep-Wake Registry has previously been described in studies on unsupervised patient clustering [24], automatic sleep scoring [25, 26], and sleep dynamics [27].

**Fig. 1:**
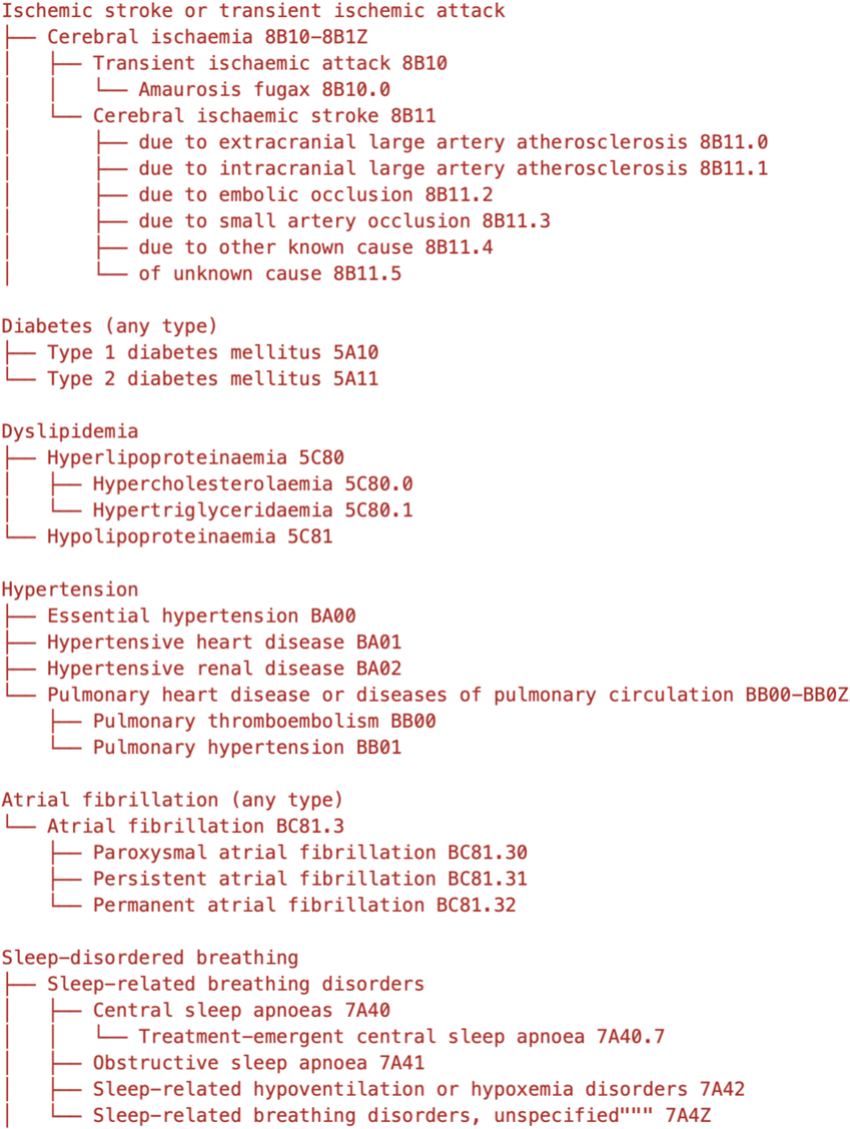
Selected comorbidity structure

To validate model performance, we created a scoring dataset consisting of 250 patient records, each containing unstructured clinical text that may describe multiple comorbidities. The 250 records were randomly sampled from the full study population (3,690) to preserve the natural distribution of documented comorbidities. Each record was independently annotated with comorbidity information by two medical doctors holding a PhD and clinical experience in sleep medicine (8 and 12 years, respectively). A third physician adjudicated disagreements. The annotation schema followed ICD–11, with subgroups as detailed in Fig. 1. A single record could carry multiple positive labels to reflect multimorbidity (e.g., concurrent hypertension and diabetes).

A key limitation of this primary dataset is its single-center origin, with documentation habits, phrasing, and clinical workflows specific to the Inselspital Bern. To obtain preliminary insight into cross-institutional robustness, we therefore constructed a secondary benchmark using 250 discharge summaries from MIMIC-IV (version 3.1). [28] Discharge summaries were selected because they most closely resemble the longitudinal, narrative-style diagnostic texts used in the Bern Sleep–Wake Registry, containing free-text descriptions of medical history, diagnoses, risk factors, and treatments rather than isolated problem lists alone. Each MIMIC-IV discharge summary corresponds to a single hospital admission and is typically associated with multiple ICD-9-CM and/or ICD-10-CM diagnosis codes, thereby reflecting patient multimorbidity. This structure makes discharge summaries well suited for evaluating document-level, multi-label comorbidity recognition. To ensure conceptual comparability across datasets and coding systems, comorbidity-related labels in MIMIC-IV were mapped to a reduced set of categories aligned with the sleep-cohort taxonomy (e.g., diabetes, hypertension, dyslipidemia, ischemic stroke, atrial fibrillation). The conceptual mapping between comorbidity categories and the ICD-9-CM / ICD-10-CM diagnosis codes used for document-level selection in MIMIC-IV is summarized in Table 7. While expert annotations in the Bern cohort followed ICD-11, weak reference labels for MIMIC-IV were derived from ICD-9-CM / ICD-10-CM codes at the category level to maintain cross-dataset consistency. Fine-grained subtypes were not evaluated for MIMIC-IV due to inconsistent reporting in ICU and discharge documentation. No additional fine-tuning, prompt adaptation, or domain-specific calibration was performed on the MIMIC-IV data; instead, the Bern-fine-tuned Mistral-24B (Instruct-2501) model was applied in a zero-shot transfer setting.

This auxiliary evaluation is not intended as a direct head-to-head comparison between sleep-medicine and ICU cohorts, but rather as a stress test of the hierarchical LLM classifier under domain, language (German vs. English), and documentation-style shift. Results are reported in Section“Results” and Table 6 in the appendix.

## Methods

**Fig. 2:**
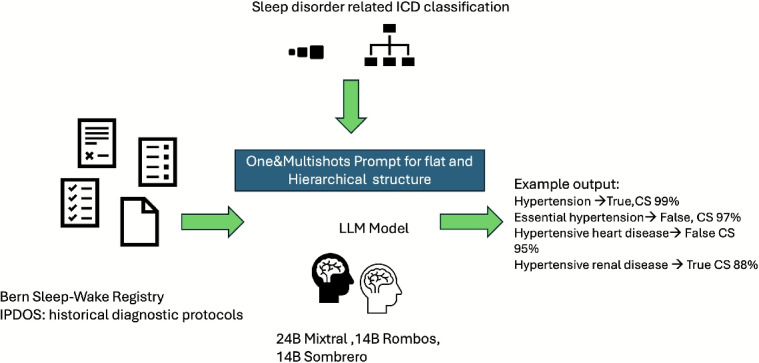
Pipeline for comorbidity extraction from historical diagnostic protocols in the Bern sleep-wake registry. Sleep-disorder related ICD categories were mapped into flat and hierarchical structures and provided as prompts to LLMs (Mistral-24B (Instruct-2501), Rombos-14B, Sombrero-14B). Model outputs include binary predictions (True/False) for each comorbidity, together with a Confidence score (CS), which are derived from token probabilities and indicate the model’s internal confidence (0–100%) in the given decision

This section details our approach to comorbidity extraction from unstructured clinical text (see Fig. 2). We first formalize the problem and notation, then present three complementary classification paradigms–flat binary, two-level hierarchical, and a LangGraph-based multi-agent variant–that operate over a selected comorbidity taxonomy. We describe prompting schemes and agent orchestration used to elicit consistent decisions, and we define objective functions that balance predictive accuracy with hierarchical consistency. Finally, we outline extraction settings, model choices, and implementation details that enable reproducible application to sleep-medicine narratives.

### Problem Setup and Notation

We formulate comorbidity extraction from unstructured clinical text as a multi-label classification task. Let:

- $x \in \mathcal {X}$: an input text (e.g., a medical report for diagnosis of sleep disorders)
- $C = \{c_1, c_2, \dots , c_n\}$: a predefined set of $n$ comorbidities organized according to the selected taxonomy

Each comorbidity $c_i$ defines the binary classification task: is $c_i$ present in $x$?

The ground truth label vector is defined as:

$$\begin{aligned} \textbf{y} = (y_1, y_2, \dots , y_n) \in \{0,1\}^n \end{aligned}$$

where

$$\begin{aligned} y_i = \left\{ \begin{array}{ll} 1 & \text {if comorbidity } c_i \text { is present} \\ 0 & \text {otherwise} \end{array}\right. \end{aligned}$$

### Model Selection

For all classification paradigms, we employ LLMs as the underlying decision engines. Three complementary instruction-tuned models are evaluated:

- **Mistral-24B (Instruct-2501)**: a state-of-the-art open-weight model with strong performance across general-purpose instruction-following benchmarks. Its larger parameter count provides high capacity for nuanced medical language understanding.
- **Rombos-14B**: a mid-sized Qwen LLM optimized for efficiency, enabling practical deployment while still maintaining competitive performance in specialized domains.
- **Sombrero-14B**: a domain-adapted variant with improved robustness on long and heterogeneous narratives, making it particularly suitable for complex sleep-medicine reports.

The combination of a large-capacity model (Mistral-24B) and two efficient 14B-parameter models (Rombos-14B and Sombrero-14B) allows us to systematically investigate trade-offs between accuracy, interpretability, and computational cost in clinical text classification.

### Classification Paradigms

We explore three classification paradigms for framing the comorbidity extraction task using LLMs, all based on the same inputs and comorbidity taxonomy: (1) flat binary classification, (2) two-level hierarchical classification, and (3) LangGraph-based multi-agent classification.

Each paradigm relies on the same input text and predefined taxonomy but differs in how predictions are structured and dependencies between categories are handled. The flat approach treats each comorbidity as an independent binary decision, the two-level hierarchical approach enforces conditional branching between parent and child categories, and the LangGraph multi-agent approach generalizes this hierarchy into a modular graph of interacting LLM agents.

#### Flat Binary Classification

For each comorbidity $c_i$, define a prompt:

$$\begin{aligned} {\text {Prompt}}(x, c_i) = \text {``Is comorbidity } c_i \text { present in the following medical text?'' } + x . \end{aligned}$$

The LLM returns a textual response:

$$\begin{aligned} r_i = {\text {LLM}}\!\big ({\text {Prompt}}(x, c_i)\big ). \end{aligned}$$

Map this response to a binary prediction $\hat{y}_i \in \{0,1\}$ via:

$$\begin{aligned} \hat{y}_i&= \left\{ \begin{array}{ll} 1, & \text {if } r_i \in \mathcal {T}_{\text {pos}},\\ 0, & \text {if } r_i \in \mathcal {T}_{\text {neg}}, \end{array}\right. \\ {\text{ where }}\\ \mathcal {T}_{\text {pos}}&= \{\text {``True''}, \text {``Yes''}, {``\_\_\mathrm{YES}\_\_''}\},\\ \mathcal {T}_{\text {neg}}&= \{\text {``False''}, \text {``No''}, {``\_\_\mathrm{NO}\_\_''}\}. \end{aligned}$$

#### Two-Level Hierarchical Classification

Let the comorbidities be organized into a two-level hierarchy:

- **Level 1 (Top-level categories)**:
  $$\begin{aligned} C^{(1)} = \{c^{(1)}_1, \dots , c^{(1)}_m\}. \end{aligned}$$
- **Level 2 (Subcategories)**:
  $$\begin{aligned} C^{(2)}_i = \{c^{(2)}_{i1}, \dots , c^{(2)}_{i n_i}\}\quad \text {for each } i \in \{1,\dots ,m\}. \end{aligned}$$

Ground-truth labels:

$$\begin{aligned} \textbf{y}^{(1)}&= \big (y^{(1)}_1, \dots , y^{(1)}_m\big ) \in \{0,1\}^{m},\\ \textbf{y}^{(2)}_i&= \big (y^{(2)}_{i1}, \dots , y^{(2)}_{i n_i}\big ) \in \{0,1\}^{n_i}. \end{aligned}$$

*Step 1:* *Top-level prediction.*
  $$\begin{aligned} r^{(1)}_i = {\text {LLM}}\!\big ({\text {Prompt}}(x, c^{(1)}_i)\big ), \qquad \hat{y}^{(1)}_i = \left\{ \begin{array}{ll} 1, & \text {if } r^{(1)}_i \in \mathcal {T}_{\text {pos}},\\ 0, & \text {if } r^{(1)}_i \in \mathcal {T}_{\text {neg}}. \end{array}\right. \end{aligned}$$
*Step 2:* *Subcategory prediction (conditional).* If $\hat{y}^{(1)}_i = 1$, then for each $j \in \{1,\dots ,n_i\}$:
  $$\begin{aligned} r^{(2)}_{ij} = {\text {LLM}}\!\big ({\text {Prompt}}(x, c^{(2)}_{ij})\big ), \qquad \hat{y}^{(2)}_{ij} = \left\{ \begin{array}{ll} 1, & \text {if } r^{(2)}_{ij} \in \mathcal {T}_{\text {pos}},\\ 0, & \text {if } r^{(2)}_{ij} \in \mathcal {T}_{\text {neg}}. \end{array}\right. \end{aligned}$$

Otherwise, set $\hat{y}^{(2)}_{ij} := 0$ for all $j$.

*Final prediction vector.* Let $d = m + \sum _{i=1}^{m} n_i$. The concatenated prediction is

$$\begin{aligned} \hat{\textbf{y}} = \big (\hat{\textbf{y}}^{(1)}, \hat{\textbf{y}}^{(2)}_{1}, \dots , \hat{\textbf{y}}^{(2)}_{m}\big ) \in \{0,1\}^{d}. \end{aligned}$$

#### LangGraph Multi-Agent Classification

LangGraph-based multi-agent classification leverages the same hierarchical taxonomy but introduces modularity via explicit computation graphs:

- **Agents:** modular LLM functions operating on ${\text {Prompt}}(x,c)$.
- **Graph:** a computation graph that routes between top-level, subcategory, and contradiction-resolution agents.

*Graph execution.*

1. **TopCategoryAgent:** produces $\hat{y}^{(1)}_i$ for each $c^{(1)}_i$.
2. **SubCategoryAgents:** if $\hat{y}^{(1)}_i=1$, activate agents for $C^{(2)}_i$ to obtain $\hat{y}^{(2)}_{ij}$.
3. **ResultJudger:** inspects $\{\hat{y}^{(1)}_i\}$ and $\{\hat{y}^{(2)}_{ij}\}$ to detect contradictions (e.g., child true but parent false).
4. **ContradictionSolver:** resolves conflicts with prompt- or rule-based logic.

*Output.*

$$\begin{aligned} \hat{\textbf{y}} = f_{\text {LangGraph}}(x; G, A) \in \{0,1\}^{d}, \end{aligned}$$

where $G$ is the graph structure and $A$ the agent library.

### Objective Functions

We use two objective functions to balance predictive performance and hierarchical validity. The Hamming loss measures overall error rate across main and subcategories, while the hierarchical consistency penalty discourages logically inconsistent predictions.

- **Hamming Loss:**
  $$\begin{aligned} \mathcal {L}_{\text {Hamming}} = \frac{1}{d}\,\sum _{i=1}^{m} \left( \textbf{1}\big [\hat{y}^{(1)}_i \ne y^{(1)}_i\big ] + \sum _{j=1}^{n_i} \textbf{1}\big [\hat{y}^{(2)}_{ij} \ne y^{(2)}_{ij}\big ] \right) . \end{aligned}$$
- **Hierarchical Consistency Penalty:**
  $$\begin{aligned} \mathcal {L}_{\text {hier}} = \sum _{i=1}^{m}\,\sum _{j=1}^{n_i}\textbf{1}\big [\hat{y}^{(2)}_{ij} = 1 \wedge \hat{y}^{(1)}_i = 0\big ]. \end{aligned}$$

### Extraction Settings

Two settings are considered for comorbidity extraction:

#### Top-level Classification

Predicts the presence or absence of each of the six main comorbidity categories:

- Ischemic stroke or transient ischemic attack
- Diabetes (any type)
- Dyslipidemia
- Hypertension
- Atrial fibrillation (any type)
- Sleep-disordered breathing

#### Two-Level Hierarchical Classification

If a main category is detected, additionally identify specific subcategories. For example:

- **Ischemic stroke**: Cerebral ischaemia, Transient ischaemic attack, etc.
- **Diabetes**: Type 1 diabetes mellitus, Type 2 diabetes mellitus
- **Sleep-disordered breathing**: Obstructive sleep apnoea, Central sleep apnoea, etc.

### Hierarchical Prompting Strategies

Designing effective prompts is central to achieving accurate and interpretable LLM predictions in hierarchical classification tasks. In this section, we outline different prompting strategies and analyze their trade-offs in terms of complexity, number of LLM calls, and suitability for various hierarchy depths (Table 1). We further illustrate how domain-specific multi-shot examples can guide the model toward consistent recognition of both top-level categories and fine-grained subtypes in sleep medicine comorbidity extraction.

**Table 1:** Comparison of hierarchical prompting strategies

Strategy	Prompt Type	# of Steps	Suitable for
Context Forwarding	Multi-step Binary	$1 + n$	Binary trees
Sequential Refinement	Binary + Multi	2	Low hierarchy
Joint Label Inference	Single-shot Multi	1	Flat classes

“# of Steps” is the number of LLM calls per document in the typical case

#### Multi-Shot Prompt Design for Comorbidity Extraction

We use multi-shot prompting with realistic examples related to sleep disorder comorbidities. The prompts are designed to teach the LLM how to recognize both top-level conditions (e.g., Sleep-disordered breathing, Cardiovascular disease) and specific subcategories (e.g., Obstructive sleep apnoea, Atrial fibrillation).

##### Agreement and Consistency Metrics

For each document *d* and parent category *p*, let the top-level agent output be $\hat{y}^{(\textrm{parent})}_{d,p} \in \{0,1\}$. Let the post-solver OR-merge of all child agents under *p* be

$$\begin{aligned} \hat{y}^{(\textrm{child,post})}_{d,p} \;=\; \max _j \hat{y}^{(\textrm{child,post})}_{d,p,j} \in \{0,1\}. \end{aligned}$$

We evaluate agreement between the parent prediction and the merged children using Cohen’s $\kappa$ and percent agreement [29], and we quantify hierarchical consistency using Hierarchical Consistency Rate (HCR) and Contradiction Resolution Efficacy (CRE).

##### Cohen’s $\kappa$ (Parent vs. Child OR)

Form the $2\times 2$ table over all (*d*, *p*) pairs:

$$\begin{aligned} \begin{array}{c|cc} & \hat{y}^{(\textrm{child,post})}_{d,p}=1 & \hat{y}^{(\textrm{child,post})}_{d,p}=0 \\ \hline \hat{y}^{(\textrm{parent})}_{d,p}=1 & a & b\\ \hat{y}^{(\textrm{parent})}_{d,p}=0 & c & d \end{array} \quad \text {with } n=a+b+c+d. \end{aligned}$$

Percent agreement is $p_o=\tfrac{a+d}{n}$ and the expected agreement is

$$\begin{aligned} p_e=\Big (\tfrac{a+b}{n}\Big )\Big (\tfrac{a+c}{n}\Big ) + \Big (\tfrac{c+d}{n}\Big )\Big (\tfrac{b+d}{n}\Big ). \end{aligned}$$

Then

$$\begin{aligned} \kappa \;=\; \frac{p_o - p_e}{1 - p_e}. \end{aligned}$$

*Hierarchical Consistency Rate (HCR)* Define a contradiction when the child OR is positive but the parent is negative. With *N* the number of (*d*, *p*) pairs,

$$\begin{aligned} \textrm{HCR} \;=\; 1 \;-\; \frac{1}{N}\sum _{d,p} \textbf{1}\!\left\{ \,\hat{y}^{(\textrm{child,post})}_{d,p}=1 \;\wedge \; \hat{y}^{(\textrm{parent})}_{d,p}=0\,\right\} , \end{aligned}$$

where contradictions are computed on *post-solver* predictions.

*Contradiction Resolution Efficacy (CRE).* If pre-solver child predictions are available, define

$$\begin{aligned} C_{\textrm{pre}}=\sum _{d,p}\textbf{1}\!\left\{ \,\hat{y}^{(\textrm{child,pre})}_{d,p}=1 \;\wedge \; \hat{y}^{(\textrm{parent})}_{d,p}=0\,\right\} ,\\\quad C_{\textrm{post}}=\sum _{d,p}\textbf{1}\!\left\{ \,\hat{y}^{(\textrm{child,post})}_{d,p}=1 \;\wedge \; \hat{y}^{(\textrm{parent})}_{d,p}=0\,\right\} . \end{aligned}$$

The efficacy of the solver is

$$\begin{aligned} \textrm{CRE} \;=\; \frac{C_{\textrm{pre}} - C_{\textrm{post}}}{C_{\textrm{pre}}} \end{aligned}$$

(Only when pre-solver predictions are logged; undefined if $C_{\textrm{pre}}=0$).

#### LangGraph ReAct Agent Workflow

**Fig. 3:**
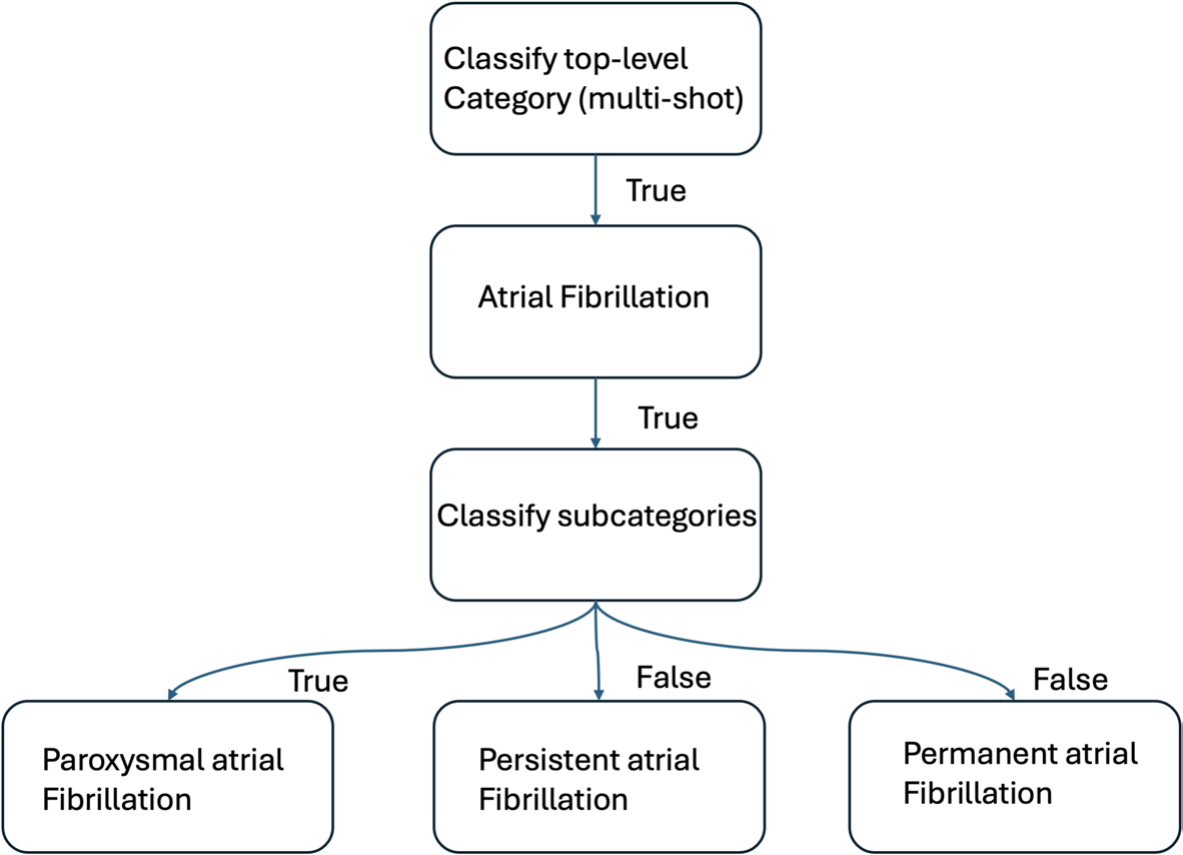
Example of LangGraph multi-shot classification

The LangChain ReAct agents in this implementation serve as modular classification tool for clinical comorbidity detection. Each ReAct agent is initialized with a specialized prompt tailored to a particular top-level comorbidity category (e.g., atrial fibrillation) or its subtypes (e.g., paroxysmal, persistent) which has been illustrated in Fig. 3. These agents take clinical text as input and produce structured True/False outputs or condition lists, depending on the classification level. By encapsulating each decision process in a separate agent, the system maintains explainability, prompt specialization, and easy extensibility–new categories or conditions can be added simply by instantiating new tools with adapted prompts.

**Fig. 4:**
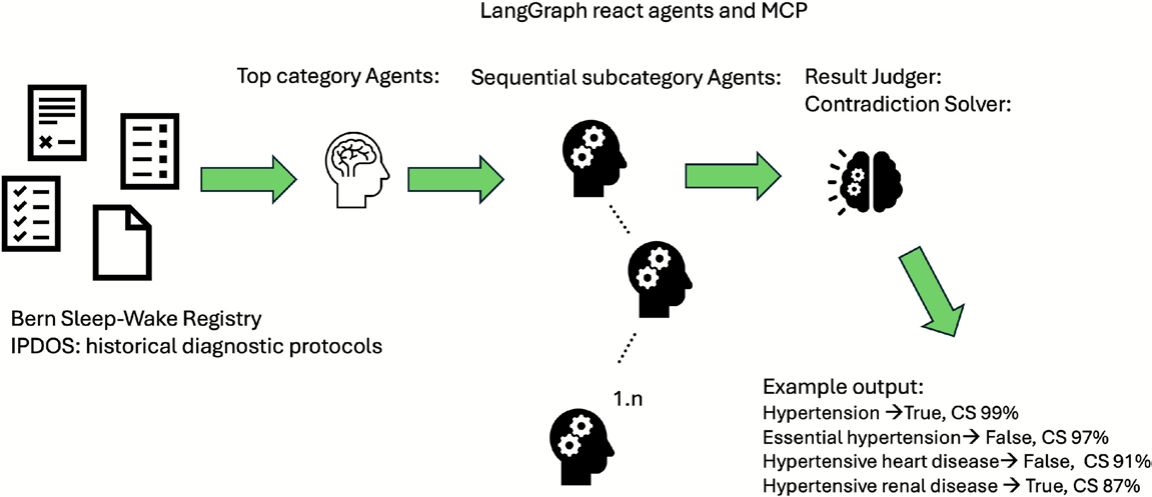
LangGraph agent workflow

In the LangGraph implementation, the overall workflow is structured into two hierarchical stages: top-level and subcategory classification. The pipeline begins by evaluating the presence of each high-level comorbidity class through a sequence of top-level agents. For any category determined as True, LangGraph conditionally routes execution to a second stage containing subcategory agents specific to that parent (see Fig. 4). Each sub-agent confirms or denies the presence of its respective condition in the clinical text while assuming the parent context is relevant. This architecture enables efficient, interpretable multi-shot reasoning with controlled branching and parallelizable decision trees, well suited for large-scale medical text classification tasks.

#### Ablation Test and Experimental Settings for Two-Level Multi-Agents

We ablate the multi-agent graph to quantify the contribution of (i) early judgment/filtering, (ii) post-hoc reconciliation of parent–child disagreements, (iii) hierarchical structure vs. a flat classifier, and (iv) parent$\rightarrow$child context forwarding. We report macro-F1 (top-level), hierarchical consistency (HCR), and cost (tokens per document), and we keep the test split, prompts, few-shot examples, and decoding parameters fixed across variants.

All runs use the same documents, the same instruction and examples, and the same base model (Mistral-24B (Instruct-2501)). For hierarchical variants we execute a parent agent for each of the six comorbidity categories and, when applicable, sub-agents for their subtypes. Agreement and consistency are computed as defined in Section“Multi-Shot Prompt Design for Comorbidity Extraction” (Cohen’s $\kappa$, HCR, CRE). Cost is measured as total prompt + completion tokens per document aggregated over all agent calls.

The following variants of settings were tested:

- Full LangGraph (baseline). Parent agents gate their corresponding sub-agents; intermediate outputs are checked by *ResultJudger*; child outputs are merged and reconciled by the *ContradictionSolver*. Parent evidence (decision + supporting snippet) is forwarded to the children (*Context Forwarding*). Final labels comprise parent predictions and post-solver child OR.
– ResultJudger. Identical to baseline except the early judgment/filter node is disabled. Downstream sub-agents execute as gated by the parent, and contradictions are still reconciled by the solver. This isolates the effect of early pruning on HCR and macro-F1.
– ContradictionSolver. Identical to baseline except the reconciliation stage is removed. Parent and child outputs are taken as-is; any parent$=$0 / child-OR$=$1 inconsistencies remain unresolved. This isolates the contribution of post-hoc consistency.
- Flat (matched cost). A single-pass, non-hierarchical classifier predicts all top-level categories and subtypes jointly (no gating, no solver, no context forwarding). To make comparisons fair, we cap the average token budget to match the baseline (*matched cost*).
- Two-Level (no solver). Hierarchical with parent gating and child sub-agents, but without the solver. Useful to separate the benefits of structural gating from those of reconciliation.
- No Context Forwarding (Atrial fibrillation (AF) subtypes). Same as baseline but the AF sub-agents receive no parent decision or evidence (no forwarding). This tests the role of parent context in fine-grained AF subtype discrimination; other categories remain unchanged.

For each variant, we compute macro-F1 at the top level, HCR from parent vs. post-solver child-OR (or n/a for the flat model), and tokens/document over all model calls. When pre-solver child predictions are logged, CRE is additionally reported to quantify the fraction of contradictions eliminated by the solver.

### Evaluation Metrics

Model performance was evaluated using accuracy, precision, recall, and F1 score, reported as both macro- and micro-averaged metrics across comorbidity classes. Macro-averaged metrics were computed by calculating each metric independently for every comorbidity class and then averaging them with equal weight, thereby reflecting per-class performance and sensitivity to class imbalance. Micro-averaged metrics were computed by aggregating true positives, false positives, and false negatives across all classes prior to metric calculation.

## Results

Appendix Table 5 presents the performance of the fine-tuned Mistral-24B (Instruct-2501) model across the six primary comorbidity categories. The model achieved consistently high accuracy across most categories, with the highest scores observed for AF (any type) (Accuracy = 0.99, Precision = 1.00, F1 = 0.96) and Diabetes (any type) (Accuracy = 0.99, F1 = 0.95). Performance for Dyslipidemia and Hypertension was slightly lower, mainly due to a drop in recall despite perfect precision in some cases, indicating conservative classification behavior. The lowest recall was observed in Sleep-disordered breathing (Recall = 0.80), reflecting the category’s clinical heterogeneity and diverse documentation styles.

Table 2 breaks down performance for each subcategory, including specific stroke etiologies, diabetes types, and sleep-related breathing disorders. Subcategory results reveal that fine-grained classification is more challenging, with some low-support conditions (e.g., Sleep-related hypoventilation or hypoxemia disorders, Type 1 diabetes mellitus) showing reduced recall due to limited training examples. In contrast, well-represented subcategories such as Obstructive sleep apnoea and Cerebral ischaemic stroke achieved strong F1-scores above 0.88.

**Table 2:** Two-level (parent $\rightarrow$ subcategory) performance of Mistral-24B (Instruct-2501) model on the test set

Parent	Subcategory	Precision	Recall	F1	Support
Ischemic stroke/TIA	Cerebral ischaemia	0.94	0.94	0.94	35
	Transient ischaemic attack	1	1	1	4
	Amaurosis fugax	1	1	1	2
	Cerebral ischaemic stroke	0.96	0.96	0.96	27
	due to extracranial large artery atherosclerosis	1	0.81	0.91	5
	due to intracranial large artery atherosclerosis	1	1	1	2
	due to embolic occlusion	0.9	1	0.95	9
	due to small artery occlusion	1	1	1	2
	due to other known cause	1	1	1	2
	of unknown cause	1	0.83	0.91	6
Diabetes	Type 1 diabetes mellitus	—	—	—	0
	Type 2 diabetes mellitus	1	1	1	3
Dyslipidemia	Hyperlipoproteinaemia	1	0.8	0.89	5
	Hypercholesterolaemia	1	0.87	0.93	8
	Hypertriglyceridaemia	1	1	1	1
	Hypolipoproteinaemia	—	—	—	0
Hypertension	Essential hypertension	—	—	—	0
	Hypertensive heart disease	1	0.91	0.95	11
	Hypertensive renal disease	1	1	1	1
	Pulmonary heart disease or	1	1	1	2
	pulmonary circulation				
	Pulmonary thromboembolism	1	0.67	0.8	3
	Pulmonary hypertension	1	0.85	0.93	7
Atrial fibrillation	Paroxysmal	——	—	—	0
	Persistent	1	1	1	1
	Permanent	1	1	1	1
Sleep-disordered					
breathing	Obstructive sleep apnoea	0.99	0.96	0.98	108
	Central sleep apnoeas	0.92	1	0.96	12
	Sleep-related hypoventilation/	1	1	1	1
	hypoxemia				
	Unspecified sleep-related	0.97	0.97	0.97	34
	breathing disorder				

Subcategories with very low support ($<$ 3 positive instances) should be interpreted with caution; apparently perfect metrics may not generalize.

Values are Precision/Recall/F1; **Support** is the number of positive test instances. Rows with zero support show metrics as em-dashes, since some subcategories had no positive examples, hence no metrics. Rows with very low support may yield inflated precision/recall

Table 3 compares macro and micro averages across four LLM architectures, demonstrating that Mistral-24B (Instruct-2501) achieved the best overall macro-F1 (0.92) and macro-precision (0.96). While competing models such as Rombos-LLM-V2.6-Qwen-14b achieved comparable accuracy, they showed slightly lower recall, suggesting that Mistral-24B (Instruct-2501) better balances sensitivity and specificity. In the single-label, mutually exclusive evaluation setting was used, micro-averaged precision, recall, and F1 are mathematically equivalent to overall accuracy.

**Table 3:** Macro- and micro-averaged metrics across four LLMs on the test set

Metric	Mistral-24B	Rombos-LLM	Sombrero-Opus	Rombos-LLM
	Instruct-2501	v2.6 Qwen-14B	14B-Elite5	v2.5 Qwen-32B
Macro Accuracy	**0.950**	0.947	0.945	0.932
Macro Precision	**0.962**	0.948	0.943	0.951
Macro Recall	0.895	**0.897**	0.896	0.853
Macro F1	**0.920**	0.919	0.917	0.891
Micro Precision	**0.950**	0.947	0.945	0.932
Micro Recall	**0.950**	0.947	0.945	0.932
Micro F1	**0.950**	0.947	0.945	0.932

Values rounded to 3 decimals. Macro-averaged metrics were computed by first calculating precision, recall, and F1 independently for each comorbidity class and then averaging these values with equal weight across classes. Micro-averaged metrics were computed by aggregating true positives, false positives, and false negatives across all classes prior to metric calculation, thereby weighting classes proportionally to their prevalence. Because comorbidity labels were evaluated in a mutually exclusive, single-label-per-document setting, micro-averaged precision, recall, and F1 are mathematically equivalent to overall accuracy. (This equivalence does not hold for multi-label settings.)

**Fig. 5:**
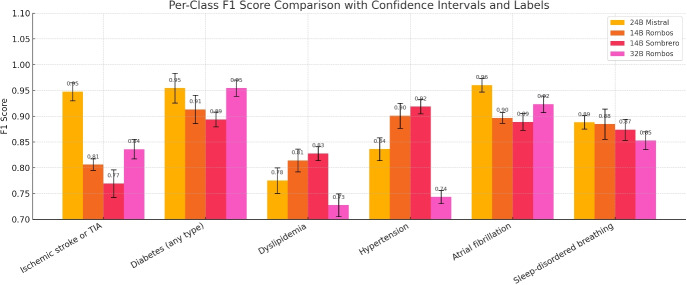
The per-class performance of models on six comorbidity classes

To probe cross-institution performance, Table 6 in the appendix shows a preliminary benchmark on MIMIC-IV discharge summaries. As expected, model performance decreases compared to the Bern cohort, reflecting domain and documentation shift. Figure 5 visualizes the per-category F1-scores as a bar chart, highlighting performance variation between categories. Stroke-related categories generally exhibited the highest F1-scores, while categories with more diffuse or variably reported clinical features, such as *Dyslipidemia* and *Sleep-disordered breathing*, exhibited greater variability.

**Fig. 6:**
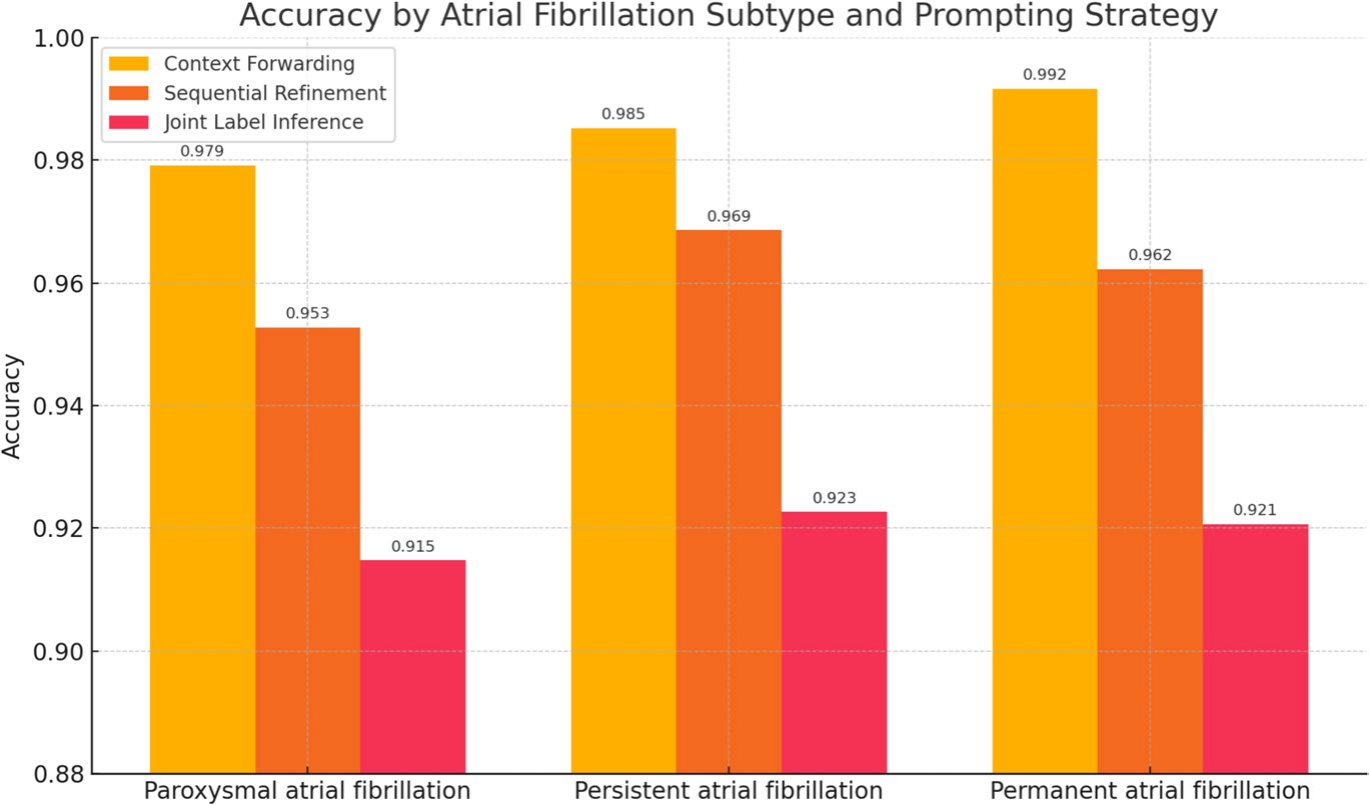
Two-level hierarchical classification: per-class metrics of models on six top comorbidity classes and 15 subcategories with different multi-shot prompting strategies using Mistral-24B (Instruct-2501)

Figure 6 compares the accuracy of three multi-shot prompting strategies–Context Forwarding, Sequential Refinement, and Joint Label Inference–across the three AF subtypes: Paroxysmal, Persistent, and Permanent. The results clearly indicate that Context Forwarding consistently achieves the highest accuracy for all subtypes, peaking at 0.992 for Permanent AF. Sequential Refinement ranks second in all cases, with accuracy values ranging from 0.953 to 0.969, while Joint Label Inference performs the weakest, with values between 0.915 and 0.923.

The ablation analysis (Table 4) shows that each component of the multi-agent pipeline contributes distinctly to both accuracy and hierarchical consistency. Removing *ResultJudger* increases child-positive/parent-negative conflicts, lowering HCR ($\approx$ -0.041) with a modest macro-F1 drop ($\approx$ -0.012). Eliminating *ContradictionSolver* degrades consistency even further (HCR $\approx$ -0.061; macro-F1 $\approx$ -0.021), confirming its role in reconciling residual disagreements between parent and sub-agents. Simpler baselines reduce compute but at a cost to performance: the *Flat* variant cuts tokens per document ($\approx$ -350) yet loses macro-F1 ($\approx$ -0.017) and offers no hierarchical notion of consistency, while the *Two-Level (no solver)* setup saves tokens ($\approx$ -180) but still lowers HCR ($\approx$ -0.028). Finally, disabling *Context Forwarding* yields small cost savings ($\approx$ -120 tokens/doc) but reduces macro-F1 ($\approx$ -0.006) and HCR ($\approx$ -0.008), mirroring the 2-8 pp accuracy declines observed for AF subtypes in Fig. 6 (largest for Persistent AF). Overall, the Full LangGraph configuration offers the best trade-off, sustaining macro-F1 and maximizing hierarchical consistency at comparable cost.

**Table 4:** Ablations: absolute metrics and deltas ($\Delta$) vs. Full LangGraph baseline

Variant	M-F1	$\Delta$ M-F1	HCR	$\Delta$ HCR	tok/doc	$\Delta$ tok/doc
Full LangGraph (baseline)	0.923	0.000	0.982	0.000	2400	0
– ResultJudger	0.911	$-0.012$	0.941	$-0.041$	2410	$+10$
– ContradictionSolver	0.902	$-0.021$	0.921	$-0.061$	2415	$+15$
Flat (matched cost)	0.906	$-0.017$	—	n/a	2050	$-350$
Two-Level (no solver)	0.912	$-0.011$	0.954	$-0.028$	2220	$-180$
No Context Forwarding (AF subtypes)	0.917	$-0.006$	0.974	$-0.008$	2280	$-120$

Notes: AF subtype accuracy drops by 2–8 pp without Context Forwarding, largest for Persistent AF. HCR is not applicable to the Flat variant (no hierarchy).

Baseline: Macro-F1 $=0.923$, HCR $=0.982$, tokens/doc $=2400$

## Discussion

Our results show that a fine-tuned Mistral-24B model, combined with a hierarchical multi-shot prompting scheme, achieves strong accuracy and F1 scores for extracting comorbidities from heterogeneous diagnostic narratives in patients presented at the tertiary sleep center.

Beyond achieving performance comparable to metrics reported in prior rule-based, classical machine learning, and transformer-based clinical NLP studies [3–5, 30], we emphasize that these references are cited for contextual comparison rather than as direct baselines, as differences in datasets, label definitions, and evaluation protocols preclude head-to-head comparison.

In addtion, the framework preserves interpretability by decomposing decisions into modular agents aligned with a clinically meaningful taxonomy–an asset for workflows that require transparent, auditable reasoning.

### Method Comparison

We observed three recurring factors behind weaker performance on Dyslipidemia and Hypertension: lexical variability and abbreviations (e.g., “HTN”, “arterielle Hypertonie”, medication proxies like “on ACEI/statin”) that are missed without explicit coverage; negation and temporality (e.g., “no history of hypertension”, “previously hypercholesterolaemic, now controlled”), which induce false positives/negatives when cues are distant; and hierarchical gating, where child conditions (e.g., hypertensive heart/renal disease) are detected but the parent label is suppressed if not explicitly mentioned. We addressed these by augmenting few-shot prompts with synonym/abbreviation lists, adding negation/temporality instructions and counter-examples, and relaxing the parent–child rule to allow parent inference when any child is confidently positive. Post-hoc, per-class thresholds further improved calibration for these categories. In addition, future work could leverage structured clinical variables such as lipid panels and blood pressure measurements to mitigate variability and improve sensitivity for dyslipidemia and hypertension.

The relatively lower F1 scores achieved by the fine-tuned Mistral-24B (Instruct-2501) model for Dyslipidemia and Hypertension likely reflect the interaction of several factors. Both conditions are highly prevalent in sleep medicine cohorts, which means the model faces greater variability in phrasing and context, including indirect mentions through medications or lab values (e.g., statins, “elevated blood pressure”) and ambiguous cases such as family history or rule-outs. This lexical and semantic diversity can make binary classification harder, especially when compared to rarer comorbidities with more explicit documentation such as stroke or atrial fibrillation. In addition, the decision thresholds learned during fine-tuning may not optimally balance precision and recall for these frequent but heterogeneous conditions, leading to more false positives or false negatives. Finally, other models like Rombos and Sombrero appear better tuned to capture cardiovascular and metabolic terminology, suggesting that domain-specific vocabulary coverage in the training data also contributes to the observed performance gap. The performance gap is most pronounced for Persistent AF, where Context Forwarding outperforms Joint Label Inference by more than six percentage points. These findings suggest that providing the model with explicit contextual information from the parent-level classification step enhances subtype discrimination, particularly for clinically subtle distinctions such as between Persistent and Permanent AF. In contrast, Joint Label Inference appears more prone to confusion when all subtype options are presented simultaneously without intermediate contextual grounding. Overall, these results confirm that the proposed hierarchical and multi-agent classification framework can achieve high accuracy while maintaining interpretability, even for clinically complex multi-label classification tasks in sleep medicine.

The hierarchical scheme reduces false positives in subcategory detection by conditioning on validated parent labels and enables more granular patient summaries. At the same time, performance differences across groups remain: For example, while ischemic stroke, diabetes, and atrial fibrillation achieved near-ceiling performance. A likely cause is that these comorbidities are often “hidden” in documentation–conveyed indirectly through proxy phrases, therapies, or metrics rather than explicit diagnoses (e.g., “PAP therapy since 20XX” or “AHI 15/h” both strongly imply sleep-disordered breathing). This variability and overlapping terminology underscore the need to (i) enrich prompts for main categories with illustrative subcategory examples to anchor model expectations, and (ii) integrate contextual cues from structured EHR data (e.g., medications, devices, procedures, quantitative indices) to surface implicit evidence and improve sensitivity. These results highlight that while the hierarchical LLM framework is capable of zero-shot transfer to new institutions, robust deployment will require multi-center training data or explicit domain adaptation to account for documentation variability. Nevertheless, the MIMIC-IV results provide a valuable stress test of the model’s generalizability and demonstrate the feasibility of using LLM-based comorbidity extraction across heterogeneous clinical environments.

Across the prompting strategies–Context Forwarding, Sequential Refinement, and Joint Label Inference–Context Forwarding yielded the best accuracy, especially for subtype tasks such as atrial fibrillation, by carrying high-level context into downstream prompts. Sequential Refinement was competitive but more sensitive to sparse notes, whereas Joint Label Inference degraded when multiple subtypes were queried simultaneously, suggesting cognitive load and weaker anchoring to prior context.

The ablation study (Table 4) quantifies how each component of the multi-agent graph affects accuracy, hierarchical consistency, and cost. Removing ResultJudger lowers macro-F1 by 0.012 (0.923$\rightarrow$0.911) and HCR by 0.041 (0.982$\rightarrow$0.941), with a small cost increase of +10 tokens per document, indicating that early filtering prevents child-positive/parent-negative contradictions from accumulating. Removing ContradictionSolver has the strongest impact on consistency, reducing macro-F1 by 0.021 (to 0.902) and HCR by 0.061 (to 0.921) while slightly increasing cost (+15 tok/doc), confirming that post-hoc reconciliation is essential for converting residual disagreements into consistent decisions. Simpler baselines reduce compute but trade off performance: the Flat (matched cost) variant saves $\approx$350 tokens per document (2400$\rightarrow$2050) yet drops macro-F1 by 0.017 (to 0.906) and lacks a meaningful HCR notion, while the Two-Level (no solver) variant saves $\approx$180 tokens (to 2220) but still reduces HCR by 0.028 (to 0.954) and macro-F1 by 0.011 (to 0.912). Disabling Context Forwarding for AF subtypes yields modest savings (-120 tok/doc) but decreases macro-F1 by 0.006 (to 0.917) and HCR by 0.008 (to 0.974), in line with the observed 2–8 percentage point accuracy declines for AF subtypes when parent context is not supplied. Overall, the Full LangGraph configuration offers the best balance, sustaining higher macro-F1 and maximizing hierarchical consistency at comparable cost.

Confidence analysis revealed cases where correct predictions were accompanied by modest confidence, indicating reliance on surface cues. Confidence-based triage is therefore useful: routing low-confidence or borderline cases to human review can increase safety without overwhelming clinicians, while high-confidence predictions can be auto-accepted to streamline throughput. Embedding multi-shot prompting within a LangGraph/ReAct agent architecture improved robustness and enabled controlled branching: top-level agents trigger only relevant subcategory agents, limiting unnecessary queries and error propagation. The modular design scales naturally–new comorbidities are added by defining targeted prompts and inserting agents into the graph without retraining the core model.

### Clinical Validation and Interpretability

The evaluation presented in this study is computational in nature and was conducted on prospectively scored comorbidity annotations. As a first study of its kind within this dataset, it is intended to lay the methodological foundation and provide initial evidence to inform future large-scale investigations. For a clinical decision-support tool, prospective clinical validation is essential. We envision a follow-up study where sleep-medicine specialists review the model’s outputs–comorbidity labels, confidence scores, and textual evidence snippets–on a sample of new diagnostic reports. Such a study would quantify not only accuracy but also perceived relevance, trustworthiness, and integration burden in clinical workflows. While the current LangGraph multi-agent architecture offers technical interpretability (e.g., clear separation between parent and child decisions, explicit reasoning steps), clinical interpretability requires that outputs be easily verifiable: a physician should be able to see *why* a comorbidity was inferred via a small number of highlighted phrases and an intuitive confidence indicator. Incorporating uncertainty quantification and calibrated confidence thresholds can support triage, where low-confidence cases are routed to human review and high-confidence cases can be processed semi-automatically.

### Clinical Use Cases and Impact

of comorbidity extraction lies in specific clinical applications. We identify several high-impact use cases in sleep medicine:

- **Rapid cohort identification** for retrospective and prospective research, such as identifying patients with both obstructive sleep apnoea and cardiovascular comorbidities.
- **Pre-screening for clinical trials**, where inclusion and exclusion criteria often hinge on the presence or absence of particular comorbidities (e.g., atrial fibrillation, recent stroke).
- **Augmenting initial diagnostic workups**, by generating concise, automatically derived comorbidity summaries for patients referred to sleep clinics, potentially saving clinician time during first visits.
- **Population health surveillance**, facilitating monitoring of multimorbidity patterns in sleep-disorder cohorts and informing resource planning.
- **Medical specialty overarching patient care**, supporting coordinated, interdisciplinary treatment plans that address the complex needs of multi-morbid patients and improve overall outcomes.
- **Multimodal phenotyping**, where text-derived comorbidity profiles are integrated with PSG, MSLT, wearable, or laboratory data to derive richer patient phenotypes and support risk stratification. In the Bernese Sleep-Wake Registry, the availability of long-term data on comorbidities and raw signals from the objective sleep assessments enables the “Sleep and Longevity” project, focusing on long-term follow-up and state-of-the-art characteristics of sleep microarchitecture [31].

### Cross-Institution Generalizability

A central limitation of this study is the use of a single-center dataset from the Inselspital Bern Sleep–Wake–Epilepsy Center for model training and evaluation. Although the 250 Bern records were randomly sampled from the full Bern Sleep–Wake Registry (study) population with available IPDOS free-text diagnostic documentation (3,690), thereby preserving the natural distribution of documented comorbidities, documentation style, phrasing, and clinical workflows remain specific to the Bern clinical environment. This center-specific context raises questions regarding the generalizability of the learned representations to other institutions.

In particular, at Inselspital Bern, especially for patients with stroke–comorbidities and risk factors are frequently documented collectively within dedicated risk-factor sections or embedded in broader diagnostic narratives, rather than being listed as separate, explicitly labeled diagnoses. This center-specific reporting practice may implicitly condition the model to rely on clustered or contextualized descriptions of comorbidity evidence.

To assess cross-institution robustness, we evaluated the Bern-fine-tuned Mistral-24B (Instruct-2501) model on 250 MIMIC-IV discharge summaries without performing any additional fine-tuning or domain adaptation. As shown in Table 6, performance decreases when the model is applied to the MIMIC-IV notes (macro-F1: 0.92 on Bern vs. 0.88 on MIMIC-IV). This reduction is driven primarily by lower recall, suggesting that the model is less sensitive to comorbidity evidence expressed in U.S. ICU-style documentation, which differs substantially in narrative structure, vocabulary, and clinical emphasis from sleep-medicine reports used during training.

Additional limitations beyond single-center bias and class imbalance include residual sensitivity to negation and temporality despite prompt engineering, as well as the lack of real-time data integration. The current model operates on static clinical notes without explicit temporal alignment to physiological signals or longitudinal disease trajectories. Addressing these limitations through multi-center training data, explicit domain adaptation, and clinician-in-the-loop evaluation will be important steps toward robust and safe clinical deployment.

## Conclusion

This study introduces a robust, interpretable, and clinically applicable framework for extracting comorbidities from unstructured sleep diagnostic texts using a fine-tuned Mistral-24B (Instruct-2501) large language model. By employing a hierarchical classification strategy and LangGraph-based multi-agent orchestration, our approach achieves macro-F1 scores above 0.92 across six major comorbidity categories. These results are competitive with, and in some cases exceed, metrics reported in prior related phenotyping and extraction studies, though comparisons across different datasets and label definitions should be interpreted cautiously.

The ability to automatically and accurately extract comorbidities from historical patient records has direct implications for personalized care, risk stratification, and research in sleep medicine. Future work should focus on expanding the taxonomy to include rarer or emerging comorbidities, modeling clinical trajectories such as the date of first symptom and date of first diagnosis, and integrating multimodal inputs including PSG signals and laboratory results. In addition, we plan to integrate structured EHR signals (medications, vitals, problem lists) for hybrid reasoning, refine calibration with class-specific thresholds and temperature scaling, and extend to multimodal inputs such as PSG summaries to reduce ambiguity in text-only notes. Cross-lingual evaluation remains an important next step to ensure generalizability across multilingual clinical environments. Furthermore, incorporating uncertainty quantification could enhance trustworthiness and guide physician review in borderline cases. Finally, developing and releasing an open-source toolbox that can be readily deployed in clinical infrastructures would accelerate translation into practice and facilitate broader adoption. Overall, our findings suggest that LLM-driven comorbidity extraction provides a reliable foundation for advanced clinical decision support in sleep medicine, representing an important step toward transforming unstructured documentation into actionable patient insights.

## Data Availability

Access to the Bern Sleep Data Base is restricted to data privacy

## Appendix B Additional Results

**Table 5:** Top-level comorbidity performance of Mistral-24B (Instruct-2501) on the test set

Comorbidity (top-level)	Accuracy	Precision	Recall	F1	Support
Ischemic stroke or transient ischaemic attack	0.984	0.923	0.973	0.947	37
Diabetes (any type)	0.992	0.955	0.955	0.955	22
Dyslipidemia	0.927	1.000	0.633	0.775	49
Hypertension	0.919	1.000	0.718	0.836	71
Atrial fibrillation (any type)	0.996	1.000	0.923	0.960	13
Sleep-disordered breathing	0.883	0.991	0.804	0.888	143

Values are per-class Accuracy, Precision, Recall, and F1; **Support** is the number of positive test instances

**Table 6:** Cross-institution benchmark comparing the performance of the Mistral-24B (Instruct-2501) model fine-tuned solely on the Bern Sleep Wake Registry and evaluated on (i) the Bern test set and (ii) MIMIC-IV discharge summaries

Metric	Bern Sleep Data in German	MIMIC-IV in English
Macro Accuracy	0.95	0.89
Macro Precision	0.96	0.90
Macro Recall	0.90	0.86
Macro F1	0.92	0.88

The reduction in macro-F1 from 0.92 on the Bern test set to 0.88 on MIMIC-IV is driven primarily by a decline in recall, indicating reduced sensitivity to comorbidity evidence expressed in ICU-oriented U.S. discharge documentation, which differs substantially from the narrative structure and clinical emphasis of sleep-medicine reports.

Reported values are macro-averaged Accuracy, Precision, Recall, and F1 computed over the six top-level comorbidity categories shown in Table 5. This comparison summarizes aggregate model behavior across clinically heterogeneous conditions and highlights performance differences between in-domain German sleep-medicine reports (Bern test set) and out-of-domain English ICU-oriented discharge documentation (MIMIC-IV). The evaluation with MIMIC-IV was performed without additional fine-tuning or domain adaptation

**Table 7:** Conceptual mapping between ICD-11 codes used for expert annotation in the Bern Sleep–Wake Registry and ICD-9-CM / ICD-10-CM diagnosis codes used for document selection in MIMIC-IV

Comorbidity category	ICD-11 codes (Bern)	ICD-9-CM codes (MIMIC-IV)	ICD-10-CM codes (MIMIC-IV)
Diabetes mellitus	5A10–5A14	250.*	E10.*, E11.*, E13.*, E14.*
Hypertension	BA00–BA0Z	401.*, 402.*, 403.*, 404.*, 405.*	I10.*, I11.*, I12.*, I13.*, I15.*
Dyslipidemia	5C80–5C8Z	272.*	E78.*
Ischemic stroke / TIA	8B11–8B1Z	433.*, 434.*, 435.*	I63.*, G45.*
Atrial fibrillation	BC81	427.31	I48.*
Sleep-disordered breathing	7A20–7A2Z	327.23, 327.2*, 780.57	G47.3*

Mapping is performed at the comorbidity category level rather than exact code equivalence

## Materials Availability

No.

## Code Availability

Code Repository: https://github.com/dyhpf/comorbidityclassifier.
